# TREM-1-Linked Inflammatory Cargo in SARS-CoV-2-Stimulated Macrophage Extracellular Vesicles Drives Cellular Senescence and Impairs Antibacterial Defense

**DOI:** 10.3390/v17050610

**Published:** 2025-04-24

**Authors:** Pedro V. da Silva-Neto, Jonatan C. S. de Carvalho, Diana M. Toro, Bianca T. M. Oliveira, Juçara G. Cominal, Ricardo C. Castro, Maria A. Almeida, Cibele M. Prado, Eurico Arruda, Fabiani G. Frantz, Ana P. Ramos, Pietro Ciancaglini, Ronaldo B. Martins, Juliano C. da Silveira, Fausto Almeida, Kelen C. R. Malmegrim, Carlos A. Sorgi

**Affiliations:** 1 Departamento de Química, Faculdade de Filosofia, Ciências e Letras de Ribeirão Preto-FFCLRP, Universidade de São Paulo-USP, Ribeirão Preto 14040-901, SP, Brazil; pedrovieira@usp.br (P.V.d.S.-N.); jonatancarvalho@usp.br (J.C.S.d.C.); jucara.cominal@gmail.com (J.G.C.); anapr@ffclrp.usp.br (A.P.R.); pietro@ffclrp.usp.br (P.C.); 2 Departamento de Análises Clínicas, Toxicológicas e Bromatológicas, Faculdade de Ciências Farmacêuticas de Ribeirão Preto-FCFRP, Universidade de São Paulo-USP, Ribeirão Preto 14040-903, SP, Brazil; ricardoccastro@usp.br (R.C.C.); frantz@usp.br (F.G.F.); rb.mj@usp.br (R.B.M.); kelenfarias@fcfrp.usp.br (K.C.R.M.); 3 Departamento de Biologia Celular e Molecular e Bioagentes Patogênicos, Faculdade de Medicina de Ribeirão Preto-FMRP, Universidade de São Paulo-USP, Ribeirão Preto 14049-900, SP, Brazil; dianamota.t@gmail.com (D.M.T.); eaneto@fmrp.usp.br (E.A.); 4 Departamento de Bioquímica e Imunologia, Faculdade de Medicina de Ribeirão Preto-FMRP, Universidade de São Paulo-USP, Ribeirão Preto 14049-900, SP, Brazil; biancatmoliveira@usp.br (B.T.M.O.); fbralmeida@gmail.com (F.A.); 5 Departamento de Medicina Veterinária, Faculdade de Zootecnia e Engenharia de Alimentos-FZEA, Universidade de São Paulo-USP, Pirassununga 13635-900, SP, Brazil; maa.almeida@usp.br (M.A.A.); cibeleprado@usp.br (C.M.P.); julianodasilveira@usp.br (J.C.d.S.); 6 Programa de Pós-graduação em Imunologia Básica e Aplicada-PPGIBA, Instituto de Ciências Biológicas, Universidade Federal do Amazonas-UFAM, Manaus 69080-900, AM, Brazil

**Keywords:** SARS-CoV-2, macrophages, extracellular vesicles, TREM-1, SASP

## Abstract

The COVID-19 pandemic, caused by SARS-CoV-2, has significantly affected global health, with severe inflammatory responses leading to tissue damage and persistent symptoms. Macrophage-derived extracellular vesicles (EVs) are involved in the modulation of immune responses, but their involvement in SARS-CoV-2-induced inflammation and senescence remains unclear. Triggering receptors expressed on myeloid cell-1 (TREM-1) are myeloid cell receptors that amplify inflammation, described as a biomarker of the severity and mortality of COVID-19. This study investigated the composition and effects of macrophage-derived EVs stimulated by SARS-CoV-2 (MφV-EVs) on the recipient cell response. Our results, for the first time, show that SARS-CoV-2 stimulation modifies the cargo profile of MφV-EVs, enriching them with TREM-1 and miRNA-155 association, along with MMP-9 and IL-8/CXCL8. These EVs carry senescence-associated secretory phenotype (SASP) components, promote cellular senescence, and compromise antibacterial defenses upon internalization. Our findings provide evidence that MφV-EVs are key drivers of inflammation and immune dysfunction, underscoring their potential as therapeutic targets in COVID-19.

## 1. Introduction

The COVID-19 pandemic, caused by SARS-CoV-2, has profoundly impacted global health, with primary impacts on the respiratory system. Severe complications, including acute respiratory distress syndrome (ARDS) and multi-organ failure, often arise from an excessive and dysregulated inflammatory response during the acute infection phase [1,2,3]. This hyperinflammation state, commonly termed a “cytokine storm”, is a hallmark of severe COVID-19 and a major factor contributing to morbidity and mortality [4]. While much attention has been directed toward the acute phase of SARS-CoV-2 infection, increasing evidence indicates that some individuals experience prolonged symptoms, collectively known as “Long COVID”, regardless of vaccination status [5]. These persistent effects, characterized by fatigue, cognitive impairment, low-grade inflammation, and immune dysfunction, underscore the need to elucidate the underlying mechanisms [6].

Among the immune components that drive a robust inflammatory response during COVID-19, the triggering receptor expressed on myeloid cells-1 (TREM-1) plays a key role. Primarily expressed on neutrophils and monocytes, TREM-1 amplifies inflammation by enhancing the production of pro-inflammatory cytokines and chemokines [7]. During SARS-CoV-2 infection, TREM-1 activation exacerbates acute inflammation, contributes to tissue damage, and intensifies the cytokine storm [8,9,10]. Previously, we identified soluble TREM-1 (sTREM-1) as a biomarker of disease severity, showing a positive correlation with inflammatory mediators such as IL-6 and IL-8, neutrophil counts, and matrix metalloproteinases (MMP)-8 [11,12]. These findings underscore the pivotal role of TREM-1 in COVID-19 pathogenesis and its potential as a target for therapeutic intervention.

Emerging evidence indicates that macrophage-derived extracellular vesicles (EVs) contribute to COVID-19 pathogenesis by altering macrophage phenotype and function [13]. EVs, which are lipid bilayer-enclosed vesicles secreted by cells, mediate intercellular communication by transferring bioactive molecules, including microRNAs (miRNAs), proteins, metabolites, and lipids [14]. In the context of COVID-19, SARS-CoV-2-stimulated macrophages may release EVs carrying pro-inflammatory mediators, sustaining and amplifying inflammation. These EVs can also interact with recipient cells, triggering phenotypic and functional changes that exacerbate inflammatory pathology [13,15]. However, the specific molecular mechanisms and pathways influenced by macrophage-derived EVs during SARS-CoV-2 infection (MφV-EVs) remain largely undefined.

One potential mechanism by which MφV-EVs may contribute to COVID-19 pathogenesis is through the induction of cellular senescence in recipient cells [16,17]. Cellular senescence is a state of irreversible cell cycle arrest associated with the senescence-associated secretory phenotype (SASP), a pro-inflammatory profile characterized by the release of bioactive molecules such as IL-6, CXCL8, MMPs, and TGF-β [18]. The SASP composition varies depending on the cell type and the stimulus that triggers senescence. While initially immunosuppressive, it transitions into a pro-inflammatory state over time, contributing to chronic inflammation, tissue dysfunction, and immune dysregulation [19]. Notably, the SASP is linked to aging, age-related diseases, and persistent inflammatory conditions [17], making it a relevant factor in Long COVID pathophysiology.

This study revealed that MφV-EVs drive the overexpression of CDKN2A and TP53, hallmark senescence-associated genes, in recipient cells. Additionally, these EVs impaired antibacterial defense mechanisms and altered inflammatory responses in recipient macrophages, suggesting a link between EV-induced senescence and the immune dysfunction observed in Long COVID. We also found that the SARS-CoV-2 interaction with macrophages leads to the production of larger EVs, potentially carrying a distinct cargo, possibly enriched with molecules involved in senescence and immune modulation. These findings underscore an important molecular mechanism contributing to the broader understanding of COVID-19 pathogenesis and its long-term consequences.

## 2. Materials and Methods

### 2.1. Cell Culture and Macrophage Differentiation In Vitro

The human monocytic leukemia cell line THP-1 (BRCJ 0234) was cultured at 37 °C in a humidified atmosphere of 5% CO_2_ using complete DMEM medium (DMEM-c), which contains 4.5 g/L glucose, L-glutamine, and sodium pyruvate (Corning, Manassas, VA, USA), supplemented with 500 U/mL penicillin (Gibco, Grand Island, NY, USA), 500 μg/mL streptomycin (Gibco, Grand Island, NY, USA), 1.25 μg/mL amphotericin (Gibco, Grand Island, NY, USA), and 10% fetal bovine serum (FBS) (Gibco, Grand Island, NY, USA). Differentiation into M0 macrophages was achieved by seeding THP-1 cells at 2 × 10^5^ cells/mL in T175 cm^2^ flasks (Greiner, Monroe, NC, USA) and treating them with 25 nM phorbol 12-myristate 13-acetate (PMA) (Sigma Aldrich, St. Louis, MO, USA) for 24 h, followed by additional 24 h in PMA-free DMEM-c, totaling 48 h of differentiation [20,21]. Subsequently, the adherent M0 were then treated with 20 ng/mL interferon (IFN)-γ (R&D Systems, Minneapolis, MN, USA) and 1 ug/mL lipopolysaccharide (LPS) (Sigma-Aldrich, St. Louis, MO, USA) for 24 h to induce the classically activated macrophage (M1) phenotype [22,23,24].

Alternatively, M0 were exposed to ultraviolet radiation (UV)-inactivated SARS-CoV-2 viral particles at a multiplicity of infection (MOI) of 2 (4.0 × 10^5^ particles/mL) for 24 h, generating the MV phenotype. After polarization or stimulation, cells were washed once, and 40 mL of EV-depleted DMEM-c was added for 24 h [25]. Finally, the culture supernatants from different macrophage (Mφ) phenotypes were collected for EV isolation and characterization, categorized as Mφ0-EVs, Mφ1-EVs, and MφV-EVs.

### 2.2. Immunofluorescence for the SARS-CoV-2 N Protein and NLRP3

To visualize cells stimulated with inactivated SARS-CoV-2 particles, M0 cells (2 × 10^5^) were seeded on coverslips in 24-well plates (Corning, USA) and exposed to viral particles at an MOI of 2 for 24 h. After stimulation, culture supernatants were discarded, and cells were fixed with 4% paraformaldehyde (Sigma-Aldrich, St. Louis, MO, USA) for 20 min at room temperature. Following PBS (pH 7.4) washes, cells were permeabilized and blocked in a solution containing 0.05% saponin (Sigma-Aldrich, St. Louis, MO, USA), 1% BSA (Thermo Fisher Scientific, Waltham, MA, USA), and 5% goat serum (Abcam, Cambridge, UK) for 1 h at room temperature. Cells were then incubated with primary antibodies against NLRP3 (1:1000—AdipoGen, San Diego, CA, USA) and the SARS-CoV-2 nucleocapsid protein (1:1000—Sino Biological, Beijing, China), followed by blocking with SuperBlock™ buffer (Thermo Fisher Scientific, Waltham, MA, USA). Detection was performed using an Alexa-Fluor 594-conjugated secondary antibody (1:500—(Thermo Fisher Scientific, Waltham, MA, USA), and nuclei were counterstained with DAPI (Sigma-Aldrich, St. Louis, MO, USA). Images were acquired using a Leica TCS SP8 confocal microscope (Leica Microsystems, Wetzlar, Germany).

### 2.3. Isolation and Purification of Mφ-EVs

To purify Mφ-EVs (Mφ0-EVs, Mφ1-EVs, and MφV-EVs), 40 mL of cell culture supernatant was processed. Cell debris was removed by sequential centrifugation at 5000× *g* for 15 min, followed by 15,000× *g* for another 15 min, both at 4 °C, using a Sorvall Legend XFR centrifuge (Thermo Fisher Scientific, Waltham, MA, USA). The resulting supernatants were concentrated using a 100 kDa molecular exclusion Amicon system (Millipore, Burlington, MA, USA). The concentrated supernatants were then filtered through a 0.45 μm membrane filter (Millipore, Burlington, MA, USA), and the precipitate retained on the membrane was discarded. The filtrates were subjected to ultracentrifugation at 100,000× *g* at 4 °C for 1 h using an ultracentrifuge (Beckman Coulter, Brea, CA, USA) equipped with a 70 Ti rotor (Beckman Coulter, Brea, CA, USA). The resulting pellet, containing purified EVs, was resuspended in 1 mL of ultrapure water (MilliQ, Darmstadt, Germany) and stored at −80 °C until further analysis.

### 2.4. Characterization of Mφ-EVs by NTA, Zeta Sizer, and Transmission Electron Microscopy (TEM)

To assess the size distribution and quantify the EVs obtained from Mφ under different stimuli, the Nano Tracking Analysis (NTA) was performed using the Nanosight NS300 equipment (Malvern Instruments, Malvern, UK). Each sample was analyzed three times, with a 30-s interval between measurements, and size distribution and dispersion graphs were generated from these data. The zeta potential (ζ) of the EVs was determined using the ZetaSizer Nano ZS (Malvern Instruments, Malvern, UK) to evaluate their surface charge properties. For morphological characterization via transmission electron microscopy (TEM), EV samples were deposited onto carbon-coated electron microscopy grids (Electron Microscopy Sciences, Hatfield, PA, USA) and allowed to adsorb for 20 min. The grids were then fixed with 1% glutaraldehyde (Sigma-Aldrich, St. Louis, MO, USA) and rinsed with deionized water (Milli-Q, Darmstadt, Germany). Images were captured using a JEM-2100 transmission electron microscope (JEOL, Tokyo, Japan) operated at an accelerating voltage of 200 kV.

### 2.5. Atomic Force Microscopy (AFM)

Samples were filtered through membranes with a pore size of 2.2 μm (Millipore, Burlington, MA, USA) and stabilized by adding glutaraldehyde (Sigma-Aldrich, St. Louis, MO, USA) in a 1:1 (*v*/*v*) ratio, resulting in a final concentration of approximately 2.5%, to prevent vesicle deformation or rupture. The mixtures were homogenized, and 10 μL of each sample was deposited onto freshly cleaved mica sheets (Ted Pella, Redding, CA, USA) and left to dry at room temperature. The micrographs were analyzed using atomic force microscopy (AFM) with a Shimadzu SPM-9600 Scanning Probe Microscopy system (Shimadzu Corporation, Kyoto, Japan) operating in dynamic mode. Scanning was performed in air at 25 °C using silicon probes (Nanosensors™, Neuchâtel, Switzerland) with a resonance frequency between 324 and 369 kHz. The scan rate was maintained between 0.2 and 0.3 Hz to avoid deformation or damage to the vesicles caused by the probe tip. The cantilever spring constants were approximately 38 ± 8 N/m, and the resonance frequencies averaged 336 ± 67 kHz. Surface roughness values were calculated using Shimadzu’s SPMOffline software (Shimadzu Corporation, Kyoto, Japan), while image analysis was performed with Nanoscope image processing software (v1.40r1, Bruker Corporation, Billerica, MA, USA).

### 2.6. Phenotypic Analysis of Mφ-EVs by Flow Cytometry

Samples were stored at −80 °C, thawed, and diluted in UltraPure DNase/RNase-Free Water (Thermo Fisher Scientific, Waltham, MA, USA). Aliquots of 100 µL from the EVs suspension were transferred into tubes containing 2 µL of monoclonal antibodies conjugated to distinct fluorochromes for the identification of surface markers: TREM-1 (PE, clone #193015; R&D Systems, Minneapolis, MN, USA), CD81 (FITC, clone JS-81; BD Biosciences, Franklin Lakes, NJ, USA), CD9 (PerCP-Cy™5.5, clone M-L13; BD Biosciences, Franklin Lakes, NJ, USA), CD63 (PE, clone H5C6; BD Biosciences, Franklin Lakes, NJ, USA), Alix (1A12, PE; sc-53540, Santa Cruz Biotechnology, Dallas, TX, USA), Calnexin (AF18, Alexa Fluor^®^ 488; sc-23954 AF488, Santa Cruz Biotechnology, Dallas, TX, USA), and Annexin V-FITC (560931; BD Biosciences, Franklin Lakes, NJ, USA). The cell-specific culture medium was used as a control. The samples were incubated for 60 min in the dark at room temperature. To control for internal autofluorescence, aliquots of the Mφ-EVs suspension were incubated in the absence of monoclonal antibodies. Additionally, aliquots of monoclonal antibodies incubated without Mφ-EVs were included as internal controls for non-specific binding. After the incubation period, 200 µL of each sample was analyzed using a CytoFLEX V3-B3-R0 flow cytometer (Beckman Coulter, Brea, CA, USA) with an aspirated volume per minute control. The flow rate was set to 30 µL/min, and data acquisition was performed for 2 min per sample. Calibration microspheres (Gigamix beads, Lyon, France) with standard sizes of 100 nm and 900 nm were used to determine the size of EV populations.

### 2.7. Prophylactic Treatment of Cells with Different Populations of EVs

In a 96-well plate (Corning, Corning, NY, USA), THP-1 cells were seeded in 100 μL of DMEM-c medium at a density of 1 × 10^5^ cells/well and treated with 25 nM PMA (Sigma-Aldrich, St. Louis, MO, USA) for 24 h. This was followed by incubation in DMEM-c without PMA for an additional 24 h, totaling 48 h of differentiation. Subsequently, the cells were prophylactically treated with the different EVs populations at a concentration of 1 × 10^6^ EVs/well for 24 h. After this treatment, the wells were washed with PBS (pH 7.4 1M), and the cells were stimulated with either ultraviolet (UV)-inactivated SARS-CoV-2 (MOI 2.0; 2.0 × 10^5^ particles/well) or LPS (500 ng/mL—Sigma-Aldrich, St. Louis, MO, USA). Following stimulation, supernatants were collected and cytokine quantification was performed within 24 h.

### 2.8. Cytokines Quantification

The production of interleukin (IL)-10 (Catalog No: 555157), IL-1β (Catalog No: 557953), and tumor necrosis factor (TNF) (Catalog No: 555212) in culture supernatants was measured using an ELISA kit (DuoSet-Human—BD Biosciences, Franklin Lakes, NJ, USA) according to the manufacturer’s instructions.

### 2.9. Bacteria Killing Assay

For the bacterial killing assay, 100 µL of THP-1 cells (1 × 10^5^ cells/well) in DMEM-c were seeded in three 96-well plates (T0, T4, T8) and treated with 25 nM PMA (Sigma-Aldrich, St. Louis, MO, USA). The cells were then treated with different EVs populations at a concentration of 1 × 10^6^ vesicles/well for 24 h. After EVs treatment, the cells were incubated with *Escherichia coli* (*E. coli* O86 wild type—ATCC, Manassas, VA, USA) at an MOI of 1.0 for 90 min at 37 °C in antibiotic-free culture medium [26,27]. The plates were washed with antibiotic-containing medium, and cells in the T0 plate were lysed with 0.5% Triton X-100 (Sigma-Aldrich, St. Louis, MO, USA) to release bacteria, followed by addition of Luria-Bertani broth (BD Difco, Sparks, MD, USA) and storage at 4 °C. Continuously, the plates T4 and T8 were incubated with medium containing antibiotics for 4 and 8 h, respectively. After these incubation periods, cells were lysed as for the T0 plate. Following completion of all time points, the plates were incubated for 4 h at 37 °C with 5% CO_2_ to allow bacterial growth. MTT solution (Sigma-Aldrich, St. Louis, MO, USA) was added, and the plates were incubated for 20 min, followed by addition of 1 N HCl (Merck, Darmstadt, Germany) to stop the reaction. Absorbance was measured at 570 nm using a spectrophotometer (BioTek, Winooski, VT, USA) [26,27].

### 2.10. Protein Concentration and Proteome Membrane-Based Immunoassay of EVs

Protein concentration was measured by BCA assay (Thermo Fisher Scientific, Waltham, MA, USA) using BSA (Sigma-Aldrich, St. Louis, MO, USA) for the standard curve. Mφ-EVs (100 μL) were lysed in 5× RIPA buffer (Sigma-Aldrich, St. Louis, MO, USA) with protease inhibitors (Thermo Fisher Scientific, Waltham, MA, USA), homogenized with an IKA Ultra Turrax T10 (IKA, Staufen, Germany), and incubated on ice. Triplicates (10 µL per sample) were incubated with BCA Working Reagent for 30 min at 37 °C, and absorbance was read at 562 nm using a spectrophotometer (BioTek, Winooski, VT, USA). The total protein (65 μg) from Mφ-EVs was analyzed using the Proteome Profiler Human XL Cytokine Array Kit (R&D Systems, Minneapolis, MN, USA). Immuno-spots were imaged via ChemiDoc™ (Bio-Rad Laboratories, Hercules, CA, USA). For the analysis in ImageJ software (v1.53, National Institutes of Health, Bethesda, MD, USA), a minimum value of 20 pixel densities was considered in each spot and in all replicates to be considered as a positive-result in the analysis. Spots that did not follow those analysis parameters were excluded from annotation.

### 2.11. RNA Extraction and qPCR

RNA from Mφ-EVs and cells was extracted using TRIzol^®^ (Thermo Fisher Scientific, Waltham, MA, USA), and mRNA was isolated with the PureLink™ RNA Mini Kit (Thermo Fisher Scientific, Waltham, MA, USA). RNA quality was assessed using a Nanodrop spectrophotometer (Thermo Fisher Scientific, Waltham, MA, USA), and cDNA was synthesized with the High-Capacity cDNA Reverse Transcription Kit (Thermo Fisher Scientific, Waltham, MA, USA). Gene expression of p16, p53, and *ACTB* was analyzed by qPCR using qPCRBIO SyGreen Mix (PCR Biosystems, London, UK) and primers (Supplementary Table S1). Cycling conditions were 95 °C for 10 min, followed by 40 cycles of 95 °C for 15 s and 60 °C for 1 min. Expression levels were quantified by the 2^−ΔΔCT^ method with *ACTB* as the reference gene, on a StepOnePlus™ Real-Time PCR System (Thermo Fisher Scientific, Waltham, MA, USA).

### 2.12. Flex Real-Time PCR for miRNA

For miRNA RT-PCR, the reverse transcription reaction was performed using the High-Capacity cDNA Reverse Transcription Kit (Thermo Fisher Scientific, Waltham, MA, USA) according to the manufacturer’s protocol. Approximately 10 ng of the total RNA was used for cDNA conversion for each target miRNA. The expression analysis utilized TaqMan^®^ stem-loop reverse transcription primers and qPCR probe primer sets: bta-miR-21-5p, bta-miR-132, bta-miR-155, and bta-miR-99b (Supplementary Table S2). For the qPCR reaction, the GoTaq^®^ qPCR Master Mix (Promega Corporation, Madison, WI, USA) was used following the manufacturer’s instructions. The PCR cycling conditions were 95 °C for 10 min, followed by 45 cycles of 95 °C for 15 s and 60 °C for 60 s, concluding with a melting curve analysis. The miRNA levels were assessed in three biological replicates, and normalization was performed using the geometric mean of Ct values of three reference RNAs (miR-99b, RNU43 snoRNA, and Hm/Ms/Rt U1 snRNA) through the 2^−ΔΔCT^ method. For mRNA analysis, the reactions included the ABI TaqMan^®^ qPCR probe primer set (Cat# 4331182), targeting β-Actin (ID# Hs01060665_g1), and the TaqMan^®^ Universal Master Mix II, without UNG (Thermo Fisher Scientific, Waltham, MA, USA), performed according to the manufacturer’s protocol. qPCR was conducted using a CFX96 Real-Time System (Bio-Rad Laboratories, Hercules, CA, USA).

### 2.13. Statistical Analysis

Data are presented as tables and graphs, using GraphPad Prism^TM^ software (version 9.1—San Diego, FL, USA). Statistical analyses were performed using the Two-way ANOVA test, followed by Tukey’s post-test, for comparisons of parametric data in the different treatments. For comparisons between different stimuli, one-way ANOVA was used, followed by Tukey’s post-test. Statistical significance was considered for *p* values < 0.05. Only significant *p*-values were shown in the graphs.

## 3. Results

### 3.1. Inactivated SARS-CoV-2 Particles Modulate Macrophage Immune Response and Macrophage-Derived EVs Size

**Figure 1:**
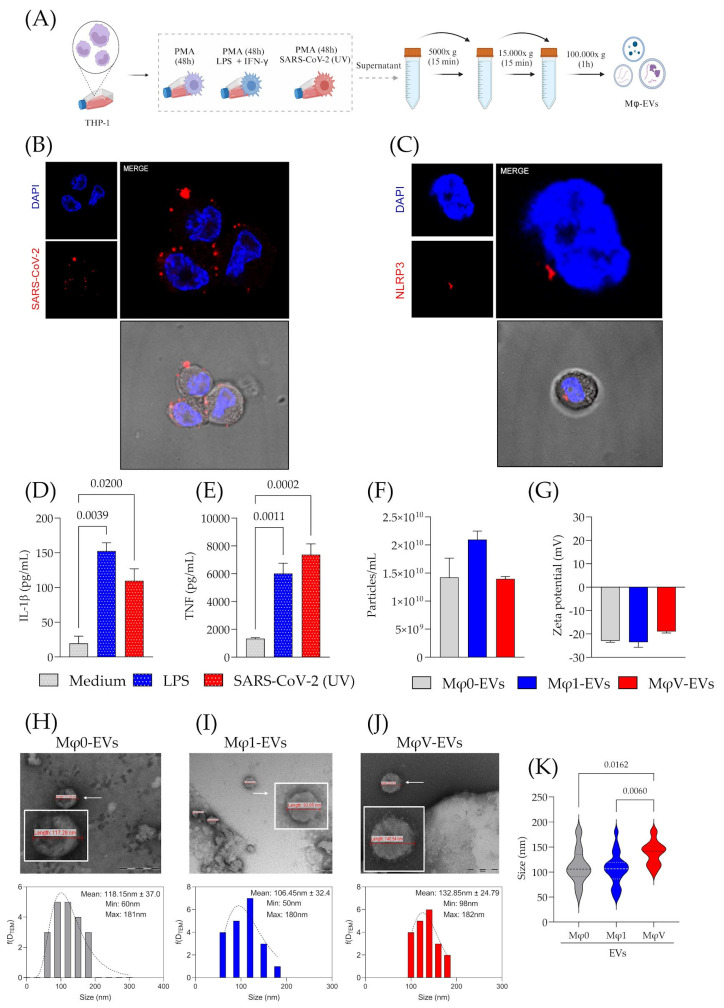
Macrophage immune response to SARS-CoV-2 particles and characterization of the released Mφ-EVs. (**A**) Workflow for Mφ differentiation and EV production and extraction. Created in BioRender (v2025, BioRender Inc., Toronto, ON, Canada—Agreement number: CK27QHOOIZ). (**B**) Representative image showing a macrophage interacting with SARS-CoV-2 particles (UV), marked in red, with nuclei stained in blue using DAPI. (**C**) Representative image of a macrophage containing NLRP3. (**D**) Production of IL-1β in Mφ culture supernatants (*n* = 4). (**E**) Production of TNF in Mφ culture supernatants (*n* = 4). (**F**) Concentration of Mφ-EVs from different macrophage stimuli measured by NTA (*n* = 3). (**G**) Electrostatic characterization (Zeta potential) of Mφ-EV membranes (*n* = 3). Morphological characterization through TEM and analysis of the size distribution by histogram of the different populations of EVs from (**H**) Mφ0-EVs, (**I**) Mφ1-EVs, and (**J**) MφV-EVs, white arrow indicated the choice vesicle to zoom view. To generate the histograms, a total of 20 pictures per group were analyzed. (**K**) Comparison of Mφ-EVs sizes from different Mφ protocols. Values were obtained from TEM image analysis. Statistical analysis was performed using a one-way ANOVA test, followed by Tukey’s post-test, considering significance at *p* < 0.05.

To generate distinct populations of Mφ-EVs, THP-1 cells were treated with different stimuli: PMA alone for the Mφ0-EVs phenotype, IFN-γ and LPS for classically activated macrophages (Mφ1-EVs), and SARS-CoV-2 inactivated by UV radiation for the viral phenotype (MφV-EVs). After differentiation and cultivation in EV-free medium, supernatants were collected for EVs extraction (Figure 1A). Immunofluorescence showed that during the 24-h stimulation, viral particles interacted with the macrophage membrane without evidence of internalization (Figure 1B). This process may have contributed to driving cell-signaling to the formation of the NLRP3 inflammasome (Figure 1C). Inflammatory cytokine analysis indicated that UV-inactivated SARS-CoV-2 significantly increased IL-1β and TNF production (Figure 1D,E) compared to controls, with no significant difference from LPS-stimulated cells (positive control). Cell viability data are shown in Supplementary Figure S1a. Previous studies have similarly used inactivated SARS-CoV-2 to induce inflammatory responses in vitro, supporting these findings [28,29,30].

Upon confirming the virus-induced inflammatory response, we proceeded to characterize the Mφ-EVs. No significant differences were observed in the concentration and zeta potential of EVs from the different Mφ populations (Figure 1F,G). Morphologically, TEM analysis showed no differences; however, histogram analysis revealed that MφV-EVs from SARS-CoV-2-stimulated macrophages were larger than those from other populations (Figure 1H–J). Statistical comparisons confirmed that MφV-EVs were significantly larger than Mφ1-EVs (*p* = 0.0060) and Mφ0-EVs (*p* = 0.0162) (Figure 1K). NTA analysis showed a size distribution within the majority of EVs between 100 and 300 nm (Supplementary Figure S2a,b). These results suggest that while the stimuli do not affect EVs production, electrical charge, or basic morphology, the interaction with the virus induces the formation of larger vesicles, which may reflect differences in their cargo.

### 3.2. Determination of Classical Markers and TREM-1 Expression in Macrophage-Derived EVs Stimulated by SARS-CoV-2 Particles

**Figure 2:**
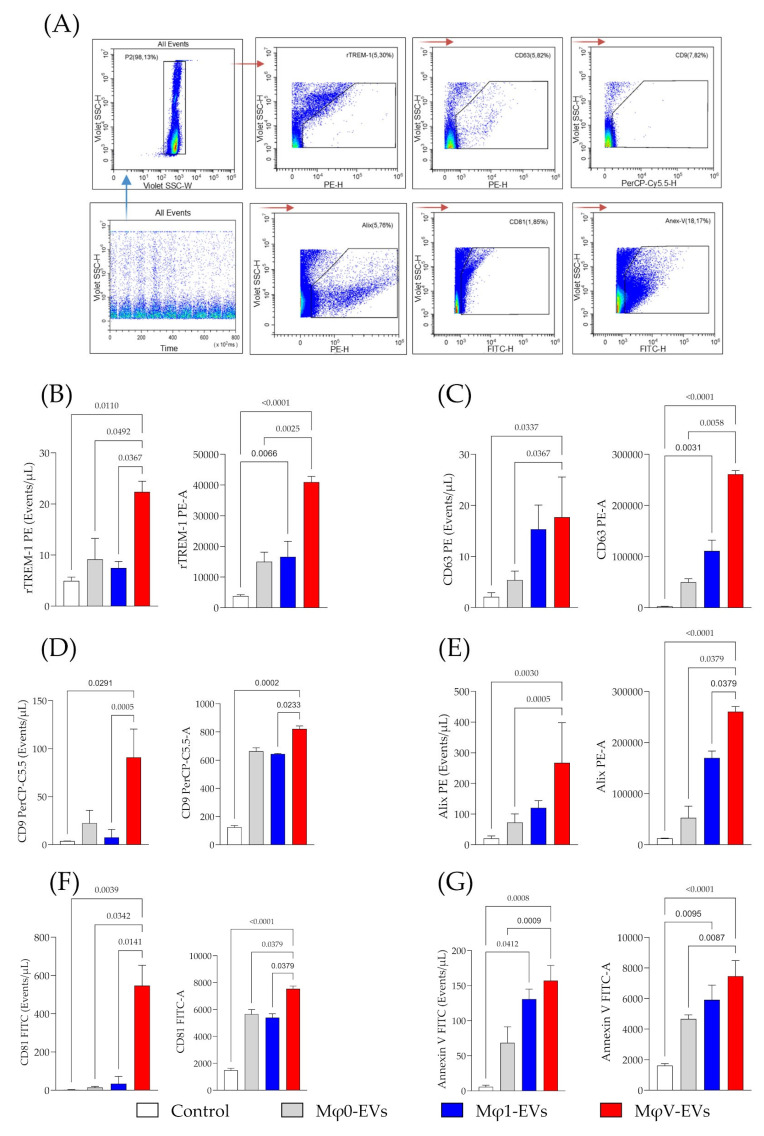
EVs derived from viral-stimulated macrophages exhibit higher TREM-1 expression and classical exosomal markers. Flow cytometry analysis of EVs. (**A**) Gating strategy for EVs identification and detection of surface markers, including TREM-1 (PE), CD63 (PE), Alix (PE), CD9 (PerCP-Cy5.5), CD81 (FITC), and Annexin V (FITC). (**B**) Detection of rTREM-1 expression in EVs derived from MφV-EVs compared to Mφ0-EVs, Mφ1-EVs populations, and Control (Medium). Expression of (**C**) CD63, (**D**) CD9, (**E**) Alix, (**F**) CD81, and (**G**) Annexin V in different Mφ-EVs populations. Data from a representative experiment out of three independent assays, conducted in a single run, showing the number of positive events/µL and distribution area—A.

Flow cytometry analysis of Mφ-EVs revealed a heterogeneous expression of surface markers across different EV populations. Specific antibodies targeting CD63, CD9, CD81, Alix, Annexin V, and TREM-1 were used to label the EVs (Figure 2A). The results confirmed the presence of classical markers in all Mφ-EVs populations, confirming their macrophage origin and typical EV characteristics. Notably, TREM-1 receptor expression, linked with inflammatory responses, was higher in EVs derived from MφV-EVs compared to Mφ1-EVs and Mφ0-EVs populations. This was evident in both the number of TREM-1-positive events and PE-A (area) (Figure 2B), indicating that SARS-CoV-2 stimulation specifically upregulated TREM-1 expression in macrophages. Furthermore, the PE-A histogram of TREM-1 expression in these EVs revealed that the increased number of TREM-1-positive events in MφV-EVs correlated with lower TREM-1 (PE-A) expression, while high-expression vesicles remained constant across all EV populations (Supplementary Figure S3a,b). Additionally, tetraspanin markers (CD63, CD9, and CD81) and Alix were more frequently detected in MφV-EVs, with intermediate levels in Mφ1-EVs and low detection in Mφ0-EVs, demonstrated by the number of positive events and area (Figure 2C–F). These results indicate a distinct molecular profile of Mφ-EVs, with increased TREM-1 expression in EVs derived from viral stimulus, suggesting functional differences based on macrophage subtype.

Annexin V was detected in both MφV-EVs and Mφ1-EVs (Figure 2G). The analysis revealed an absence of calnexin in the tested vesicles, confirming the absence of cellular contamination (Supplementary Figure S4a–c). The vesicle size distribution analyzed by CytoFLEX flow cytometer showed a homogeneous distribution among the EVs groups, with the predominant size between 100 and 300 nm in MφV-EVs (Supplementary Figure S4d). Indeed, these findings underscore the unique inflammatory signature of MφV-EVs, potentially influencing immune modulation during viral stimulation.

### 3.3. Prophylactic Treatment with EVs Modulates the Inflammatory Response and Bacterial Killing Activity of Macrophages

**Figure 3:**
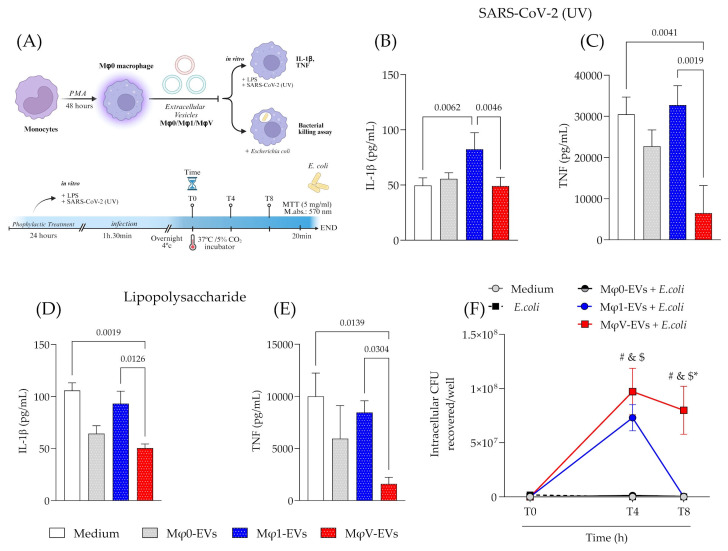
Modulation of the inflammatory response and bactericidal activity of macrophages by EVs. (**A**) Workflow of the inflammatory response assay to different PAMPs and microbicidal capacity of cells treated with EVs. Created in BioRender (v2025, BioRender Inc., Toronto, ON, Canada—Agreement number: XT27QHP2RI). Quantification of (**B**) IL-1β and (**C**) TNF in the supernatant of macrophages pre-treated with Mφ-EVs and stimulated with SARS-CoV-2 (UV) (*n* = 4). Quantification of (**D**) IL-1β and (**E**) TNF in the supernatant of macrophages pre-treated with Mφ-EVs and stimulated with LPS (*n* = 4). Statistical analyses were performed with a one-way ANOVA test, followed by Tukey’s post-test, considering significance at *p* < 0.05. (**F**) Total *E. coli* recovered after the bacterial killing assay (*n* = 4). Statistical analyses were performed with a Two-way ANOVA test, followed by Tukey’s post-test, considering significance at *p* < 0.05 for ^#^ medium vs MφV-EVs + *E. coli*; ^&^ *E. coli* vs MφV-EVs + *E. coli*; ^$^ Mφ0-EVs + *E. coli* vs MφV-EVs + *E. coli*; * Mφ1-EVs + *E. coli* vs. MφV-EVs + *E. coli*.

To assess the immunomodulatory effects of Mφ-EVs, we performed prophylactic treatment with Mφ0-EVs, Mφ1-EVs, or MφV-EVs for 24 h in macrophage culture. Cells were then stimulated with SARS-CoV-2 (UV) or LPS to evaluate cytokine production (within 24 h post-treatment) or *E. coli* infection to bacterial killing activity (Figure 3A). Cells pre-treated with Mφ1-EVs and subsequently exposed to inactivated virus showed higher IL-1β production, with no significant differences in the other groups (Figure 3B). However, TNF production was significantly reduced in cells pre-treated with MφV-EVs, compared with untreated cells (*p* = 0.0041) and cells treated with Mφ1-EVs (*p* = 0.0019) (Figure 3C). When carrying out the same evaluation for cells stimulated with LPS, it was observed that pre-treatment with MφV-EVs also reduced IL-1β and TNF production compared to untreated cells or those treated with Mφ1-EVs (Figure 3D,E). IL-10 production was also evaluated but not detected in any of the treatments. Cell viability data are shown in Supplementary Figure S1b,c. These findings indicate that EVs treatment can modulate inflammatory cytokines production in macrophages, with distinct effects depending on the EVs population.

To further explore whether EVs pre-treatment affects macrophage functions, a bacterial killing assay using *E. coli* was performed, with different incubations derived from Mφ0-EVs, Mφ1-EVs, and MφV-EVs populations. Within 4 h of bacterial internalization, cells treated with Mφ1-EVs and MφV-EVs showed reduced bacterial killing compared to cells treated with Mφ0-EVs and the control group (non-EV treatment) (Figure 3F). However, after 8 h, macrophages treated with Mφ1-EVs regained their bactericidal activity, similar to the other groups, while cells treated with MφV-EVs continued to show reduced bacterial killing (Figure 3F). These results demonstrate that MφV-EVs treatment impairs the inflammatory response and compromises the macrophage’s ability to eliminate bacterial infections efficiently.

### 3.4. EVs from SARS-CoV-2-Stimulated Macrophage Display a Distinct Proteomic Profile and Enhanced Expression of Specific miRNA

**Figure 4:**
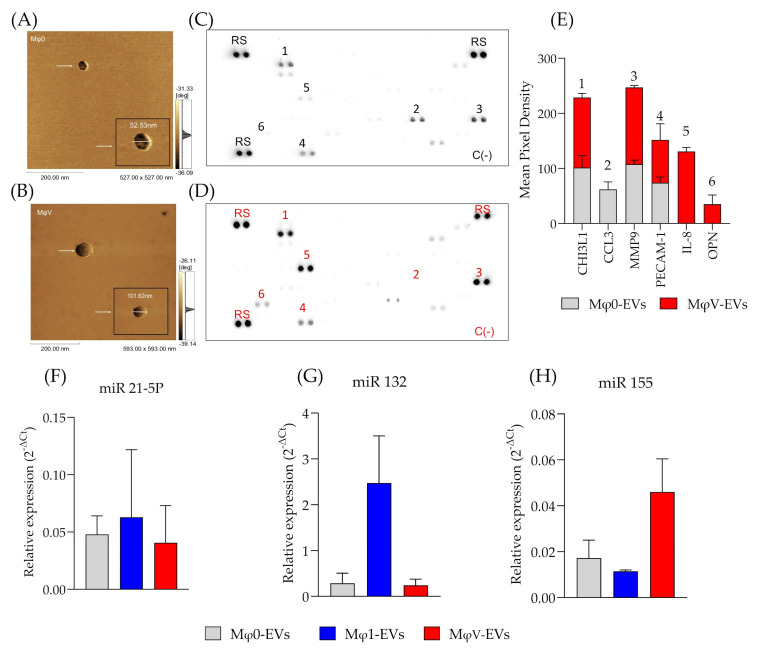
Differential abundance of proteins in different Mφ-EVs populations and miRNA cargo. (**A**) AFM image of Mφ0-EVs derived from cells treated with 25 nM PMA for 24 h. (**B**) AFM image of MφV-EVs derived from cells stimulated with UV-inactivated viral particles. Proteome analysis was performed using the Proteome Profiler Human XL Cytokine Array kit, normalized by equal amounts of total protein of (**C**) Mφ0-EVs and (**D**) MφV-EVs lysates. Representative array blots are shown after a 2-min exposure time. (**E**) Average signal intensities of spots within the matrix patches, measured across three independent experiments. Protein labels correspond to the respective numbered spots on the blots. Analysis was performed with ImageJ software, including background subtraction, with a minimum value of 20 pixel densities for all replicates. RT-qPCR analysis of miRNA expression for (**F**) miR-21-5p, (**G**) miR-132, and (**H**) miR-155 in relation to the population of Mφ0-EVs, Mφ1-EVs, and MφV-EVs. The experiment was conducted with biological triplicate with independent samples. RS = Reference Spots and C(−) = Negative Controls.

To investigate whether SARS-CoV-2 stimulation alters the loading of specific proteins and miRNAs into the TREM-1 pathway, we performed a detailed analysis. Similar to what was previously demonstrated in TEM analyses, AFM (Figure 4A,B) confirmed that MφV-EVs exhibited distinct characteristics, particularly size differences (Figure 4B). AFM phase images revealed that the surface of MφV-EVs was different from that of Mφ0-EVs, as indicated by the presence of protrusions (vesicle surface), which could suggest protein aggregation or changes in lipid composition (Supplementary Figure S5a–f). Further, we performed a comparative proteomic analysis of Mφ0-EVs and MφV-EVs across three biological replicates. The number of identified proteins was normalized by internal control (Supplementary Table S3). Quantitative analysis showed that most proteins were common across EVs populations (Figure 4A–D). However, we identified that CHI3L1, MMP-9, PECAM-1, IL-8/CXCL8, and OPN proteins were more abundant in MφV-EVs compared to Mφ0-EVs (Figure 4E). Additionally, miRNA analysis (Figure 4F–H) revealed that miRNA-155 (Figure 4H) was the most abundantly loaded miRNA in MφV-EVs compared to Mφ0-EVs. These findings suggest that EVs derived from cells stimulated with SARS-CoV-2 particles carry distinct inflammatory proteins and miRNA. This unique cargo could influence the response of recipient cells, potentially modulating their inflammatory response.

### 3.5. Treatment with MφV-EVs Markedly Affected the Expression of Genes Involved in Senescence Pathways, Particularly CDKN2A and TP53, in Macrophages

**Figure 5:**
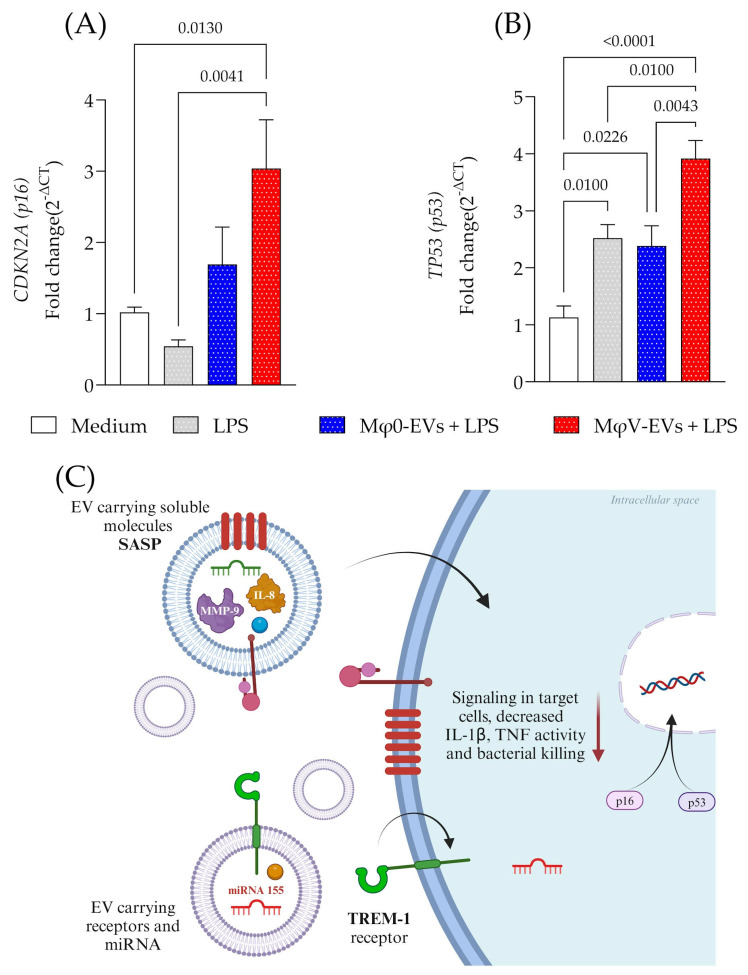
Overexpression of CDKN2A (p16) and TP53 (p53) in macrophages treated with inflammatory EVs. Gene expression of (**A**) CDKN2A (p16) and (**B**) TP53 (p53) in macrophage lysates 48 h after treatment with different Mφ-EVs. (**C**) Schematic representation of SASP induction by EVs treatment, focusing on TREM-1, miRNA, and inflammatory mediator expression. This supports the hypothesis that MφV-EVs carry early immunosenescence markers and influence SASP modulation. Additionally, miRNAs delivered by EVs may regulate the immune response and the senescence process, as evidenced by miR-155 (↑). These results correspond to independent experiments. Statistical analyses were performed using the *t*-test * *p* < 0.05. Created in BioRender (v2025, BioRender Inc., Toronto, ON, Canada—Agreement number: HE27QHOQUJ).

To investigate whether EV components influence senescence pathways, we measured the expression of CDKN2A (p16) and TP53 (p53) genes in cell lysates 48 h after treatment with Mφ0-EVs or MφV-EVs. We observed a significant increase in CDKN2A (Figure 5A) and TP53 (Figure 5B) expression genes in macrophages treated with MφV-EVs. These genes are critical regulators of cell cycle arrest and senescence. Our findings suggest that EVs (Figure 5C) released by SAR-CoV-2-stimulated cells with cargo of metalloproteinases (MMP-9), chemokines (IL-8/CXCL8), immune receptors (PECAM-1 and TREM-1), and mirRNA-155 directly impact the expression of genes associated with senescence, such as CDKN2A (p16) and TP53 (p53), thereby modulating macrophage activity. Notably, elevated expression of these markers may be associated with canonical senescence pathways, often triggered by stress stimuli, including inflammatory or viral challenges.

## 4. Discussion

SARS-CoV-2, the etiological agent of COVID-19, significantly impacts the immune system, particularly macrophages, which are crucial in coordinating immune responses. During infection, SARS-CoV-2 can modify macrophage phenotypes, resulting an imbalance in the production of inflammatory mediators. This disruption contributes to the hyperinflammatory response seen in severe COVID-19 cases, which is marked by elevated levels of cytokines such as IL-6, TNF, and IL-1β [31]. For the first time, we demonstrated that SARS-CoV-2 stimulation alters the cargo composition of macrophage-derived EVs, enriching them with inflammatory components like TREM-1 and associated miRNA-155, along with MMP-9 and IL-8/CXCL-8. These EVs, when interacting with recipient cells, activate SASP pathways, carrying pro-inflammatory molecules. Our findings showed that MφV-EVs not only modulate inflammatory responses but also disrupt normal immune functions, weakening antibacterial defense mechanisms. Additionally, the presence of miRNA-155 and other signaling molecules in MφV-EVs worsens macrophage effector dysfunction. Overall, our results showed the pivotal role of macrophage-derived EVs in the pathophysiology of SARS-CoV-2 infection, demonstrating their dual impact in driving hyperinflammation and contributing to long-term immune dysfunction in COVID-19.

Exacerbated inflammation in COVID-19 has been associated with worse clinical outcomes, often referred to as Post-COVID-19 Conditions (PCC). Despite this, the underlying mechanisms remain unclear [32]. It has been reported that cytokines like IL-1β and IL-18 contribute to fibroblast activation, leading to the synthesis and accumulation of type I collagen, TIMP, collagenase (e.g., MMPs), and PGE_2_, which may contribute to lung dysfunction in affected patients [33,34]. Additionally, some COVID-19 patients succumb to the disease despite low viral loads, exhibiting heightened inflammasome activation and increased levels of inflammatory mediators. This suggests that the uncontrolled inflammatory response may not solely depend on the virus itself but also involve Damage-Associated Molecular Patterns (DAMPs) [35]. In line with these findings, our analyses demonstrated that inactivated SARS-CoV-2 effectively stimulates IL-1β production, TNF, and NLRP3 inflammasome formation, serving as a reliable model to replicate the inflammatory response observed in COVID-19 patients [28,29,30]. Moreover, this model also mirrors the situation in vaccinated patients, where the virus is inactivated post-replication but still triggers immune responses. Recent studies have demonstrated the critical role of EVs in viral infections, including their involvement in immune evasion and inflammation during diseases like COVID-19 [36].

EVs are lipid bilayer-enclosed structures released by cells into the extracellular environment, facilitating intercellular communication through the transfer of biomolecules, including proteins, lipids, and nucleic acids. They are commonly classified into exosomes, microvesicles, and apoptotic bodies, based on their size, biogenesis, and release mechanisms [37]. The production of EVs from in vitro-activated macrophages is well-established, with reproducible protocols for their extraction and purification. Our method for obtaining macrophage-derived EVs yielded concentrations and morphology consistent with previous reports [38,39]. The size distribution and physicochemical properties aligned with reference values for microvesicles and exosomes, without evidence of aggregate formation. Additionally, we observed heterogeneity in the average size of EVs derived from macrophages with different phenotypes, where EVs secreted by macrophages stimulated with viral particles were significantly larger than those from other populations. Despite this increase in size for MφV-EVs, flow cytometry analysis confirmed that the majority of the EVs were exosomes, as indicated by the positive expression of markers such as Alix and CD9 [40,41,42]. These results suggest that the observed size variations in secreted EVs may be linked to the inflammatory cargo acquired during innate immune recognition of SARS-CoV-2, influencing the expression of miRNAs and proteins, such as TREM-1 and miR-155.

Analysis of the function of EVs secreted by the different macrophage phenotypes showed that cells treated with MφV-EVs had a reduced ability to secrete important cytokines, such as IL-1β and TNF, following stimulation with either SARS-CoV-2 (inactivated) or LPS. This reduction in inflammatory cytokine production suggests a possible impairment of the capability to eliminate pathogens [43,44]. Due to this hypothesis, a bacterial killing assay was performed, showing that cells treated with MφV-EVs were not efficient in killing *E. coli*, even after 8 h of in vitro incubation. These results align with a previous study, where dermal macrophages from older individuals showed impaired TNF production, affecting the specific immune response to *C. albicans* [45]. Therefore, EVs produced in response to SARS-CoV-2 could induce a state of immunosuppression in macrophages, characterized by reduced production of inflammatory cytokines and impairment of the ability to eliminate pathogens. This mechanism resembles senescence, a process already described in pulmonary infection by SARS-CoV-2 [46].

It is well-established that EVs play a central role in coordinating immune responses [14] by transporting molecules associated with immune activation and oxidative stress [47]. Moreover, EVs secreted by senescent cells can alter the local microenvironment, potentially inducing senescence in neighboring cells [48]. Senescent cells remain metabolically active and secrete a complex array of pro-inflammatory cytokines, growth factors, and proteases, collectively known as the SASP [49]. The pro-inflammatory environment created by SASP factors can modify the extracellular matrix, modify immune responses, and even facilitate tumor progression in certain contexts [49]. Understanding the mechanisms regulating the SASP is critical for developing therapeutic strategies to mitigate the adverse effects of senescent cells while preserving their beneficial functions. However, the SASP and EV-cargo may represent a novel mechanism for cell–cell signaling and modulation. To investigate the cargo carried by these vesicles, we performed specific protein and miRNA profiling.

SASP consists of a wide range of bioactive molecules, including pro-inflammatory cytokines (e.g., IL-1, IL-6, IL-8, TNF), chemokines (e.g., CCL2, CCL5, CXCL1), growth factors (e.g., VEGF, HGF), and proteases (e.g., MMPs). These components collectively contribute to the inflammatory microenvironment and have distinct roles in modulating immune responses and tissue homeostasis [50]. IL-8, a potent chemoattractant, facilitates the infiltration of neutrophils and promotes the degradation of extracellular matrix components through MMPs, which remodel the extracellular matrix and influence cellular communication, potentially aiding in tissue repair but also contributing to tumor invasion and inflammation in pathological contexts [51]. In our study, we observed increased amounts of IL-8/CXCL8 and MMP-9 in the MφV-EVs cargo, suggesting a potential mechanism for proteolytic regulation in the microenvironment where these EVs are generated and other regions [52,53]. MMPs have been previously associated with EVs [54], and earlier studies revealed that melanoma [55] and colorectal carcinoma [56] cells release vesicles exhibiting gelatinolytic and/or collagenolytic activities.

Interestingly, we observed a significant increase in the expression of TREM-1 and miR-155 in MφV-EVs. TREM-1 exerts pro-inflammatory effects by inducing miR-155, which regulates the expression of SOCS [57] and inositol 5′-phosphatase (SHIP)-1 [58]. We propose that the elevated levels of miR-155-5p in MφV-EVs may influence senescence pathways in recipient macrophages, as evidenced by a marked increase in CDKN2A (p16) and TP53 (p53) gene expression. During HIV-1 infection, inflammation stimulates the production of larger EVs containing a high amount of miR-155 [59], supporting previous findings that EV-miR-155 in plasma are robust biomarkers of increased viral replication [60] and immune activation [61]. Conversely, the high abundance of miR-21-5p in EVs was positively associated with metabolic markers of oxidative stress and negatively correlated with anti-inflammatory polyunsaturated fatty acids [47]. Additionally, miR-21-5p was linked to the downregulation of genes involved in inflammation and apoptosis, such as IL-6, TNF, STAT3, ICAM-1, NF-κB, cleaved caspase-3, cleaved caspase-9, and FasL [62]. These findings demonstrated the complex regulatory roles of EV-contained miRNAs in modulating inflammatory and senescence pathways.

Senescent cells can significantly impair macrophage bactericidal functions by inducing immune dysregulation through the secretion of SASP factors. Pro-inflammatory cytokines released by senescent cells can either polarize macrophages or induce exhaustion, both of which compromise their ability to phagocytose and kill bacteria effectively [63]. Additionally, the accumulation of reactive oxygen species (ROS) and other oxidative stress markers from senescent cells exacerbates macrophage dysfunction in antimicrobial activity [64]. This dysregulated response may result in an inability to resolve bacterial infections and an increased susceptibility to secondary infections, as observed in vitro with macrophages pre-treated with MφV-EVs and infected with *E. coli*. Recent advances to characterize senescence in vivo during aging or acute inflammation revealed that macrophages share phenotypic similarities with senescent cells, such as induction of p16^Ink4a^ expression [65]. These findings underscore the detrimental role of senescent cells in modulating macrophage function and their broader implications for infection susceptibility and chronic inflammation in aging tissues. Our study also demonstrated that SARS-CoV-2-stimulated macrophages can induce similar senescent effects in the neighboring cells by the release of EVs.

Thus, we provide important evidence on the mechanisms through which EVs from COVID-19 contribute to the induction of SASP, potentially influencing immune response modulation of recipient cells [66,67]. Despite the need for in vivo studies to confirm this mechanism of senescence driven by SARS-CoV-2, current evidence suggests that the virus induces cellular senescence through the EV-pathway, contributing to immune dysregulation. These processes may impair macrophage function, reduce bactericidal activity, and perpetuate a pro-inflammatory environment. Overall, SASP-EVs exert profound effects on immune response and homeostasis, balancing beneficial outcomes such as immune surveillance and detrimental consequences like chronic inflammation, tissue degradation, and the promotion of age-related diseases.

## 5. Conclusions

In summary, our findings demonstrate that SARS-CoV-2 stimulation significantly alters the profile of macrophage-derived EVs, affecting their protein composition, receptor expression, such as TREM-1, and intravesicular cargo, including miRNAs such as miRNA-21-5p and miRNA-155. These cargo profiles are associated with the modulation of key pathways linked to cellular senescence, particularly through the overexpression of senescence-related markers such as CDKN2A and TP53 in recipient cells. These EVs influence critical processes such as antibacterial defense, inflammatory response regulation, and possibly immune senescence, contributing to the broader context of immune dysregulation, which may be relevant in the context of Long COVID.

### Limitations of the Study

This study was conducted using the THP-1 cell line, and in vivo analysis was not feasible. Future studies will focus on exploring this mechanism in primary human cells and evaluating the functional effects of EVs in animal models infected with SARS-CoV-2 to validate our findings.

## Data Availability

The original contributions presented in the study are included in the article. Further inquiries can be directed to the corresponding author.

## Supplementary Materials

The following supporting information can be downloaded at: https://www.mdpi.com/article/10.3390/v17050610/s1, Figure S1: The addition of EVs did not impact cell viability during culture; Figure S2: Size distribution measured by nanoparticle tracking analysis (NTA NS3000, Malvern); Figure S3: TREM-1 expression demonstrated by the PE-A histogram of EVs-macrophage populations; Figure S4: Viral vesicles (MφV-EVs) show a high distribution of nano-sized particles detected by flow cytometry; Figure S5: AFM micrographs of Mφ0-EVs and MφV-EVs groups; Table S1: Genes analyzed by qPCR and their respective primers and transcription products for gene expression analysis; Table S2: Raw cycle threshold levels of the panel of miRNAs used in extracellular vesicle profiling; Table S3: Amount of total protein in Mφ0-EVs and MφV-EVs.
