# Supplementary material for: TREM-1-Linked Inflammatory Cargo in SARS-CoV-2-Stimulated Macrophage Extracellular Vesicles Drives Cellular Senescence and Impairs Antibacterial Defense
Source: Viruses. 2025 Apr 24;17(5):610. doi: 10.3390/v17050610 (PMC12115590; doi:10.3390/v17050610)
Supplement: Supplementary file 1 [file viruses-17-00610-s001.zip › viruses-3488694-supplementary.pdf]

Supplementary Materials

S1. Viability Assay

For the cellular cytotoxicity assay, after the treatments carried out on the culture plates, the cell supernatants were removed and replaced with 100 μL of 3-[4,5-dimethyl-thiazol-2-yl]-2,5 diphenyltetrazolium bromide solution. (MTT) (0.5 mg/mL), diluted in DMEM-c. After adding this solution, the cells were incubated for 4 h at 37ºC and 5% CO_2. Then, the culture supernatants were removed and 100 μL of dimethyl sulfoxide (DMSO) was added to the wells for 30 min. or until total solubilization of formazan crystals, originating from the mitochondrial metabolism of MTT. Absorbance measurements were obtained using a microplate spectrophotometer at 570 nm (μQuant - BioTek, Instruments, INC). Viability was defined as the ratio (expressed as a percentage) of the absorbance of treated cells relative to untreated cells.

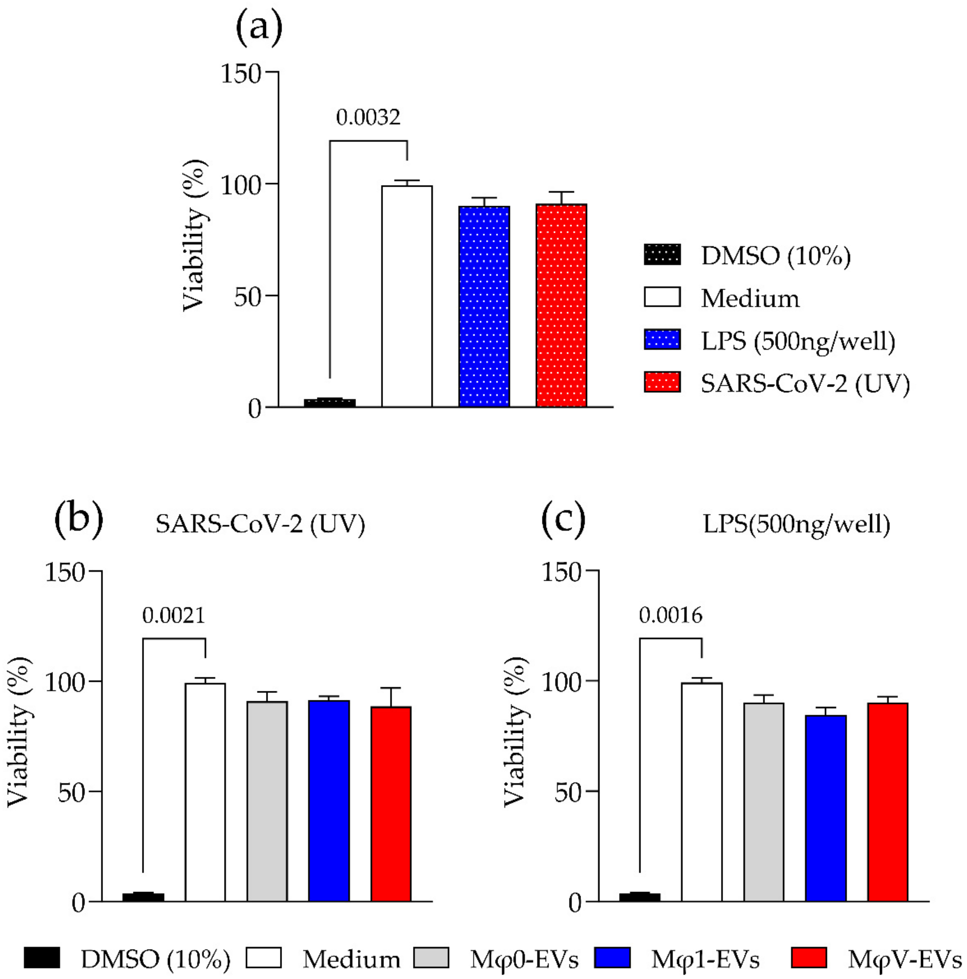

Figure S1. The addition of EV did not impact cell viability during culture. (**a**) Cell viability assay under different inflammatory stimulus (n = 4). (**b**,**c**) Cell viability assay after the experiment with prophylactic treatment, under different stimuli, SARS-CoV-2 (UV) and LPS (n = 4), respectively. Statistical analyzes were performed with one-way ANOVA test, followed by Tukey's post-test, considering significance at $p < 0.05$.

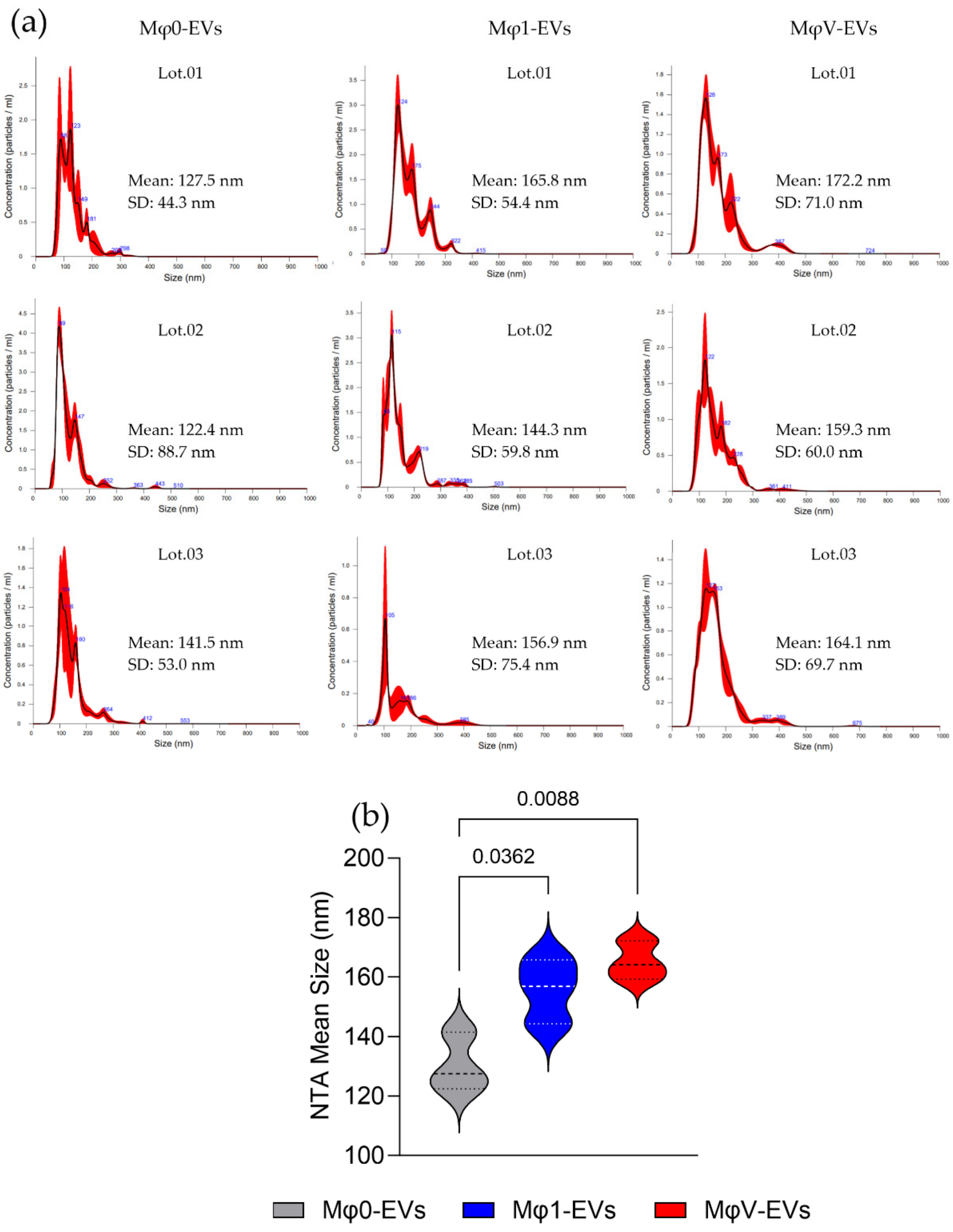

Figure S2. Size distribution measured by nanoparticle tracking analysis (NTA NS3000, Malvern). (**a**) The size and concentration of isolated EVs was analyzed using nanoparticle tracking analysis. (**b**) Comparison between NTA mean sizes from different Mφ-EVs. Statistical analyzes were performed with a one-way ANOVA test, followed by Tukey's post-test, considering significance at $p < 0.05$.

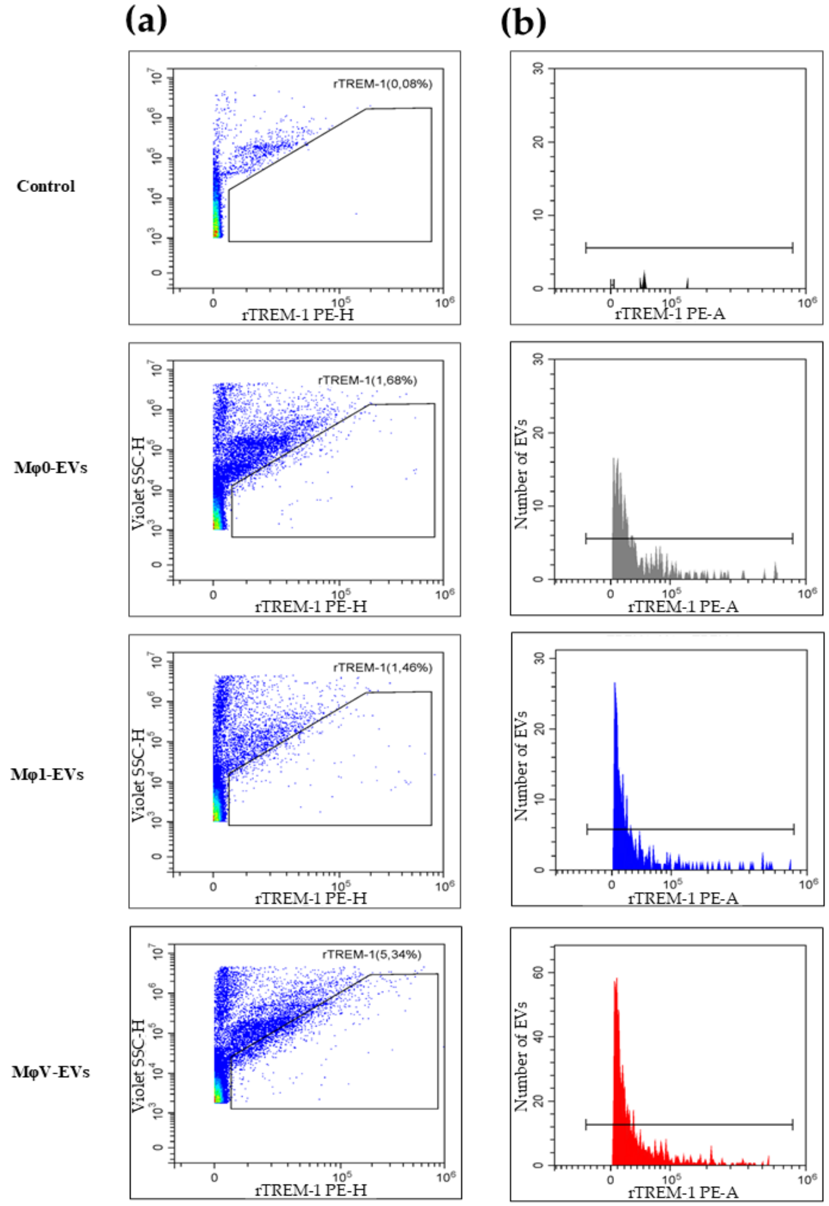

Figure S3. TREM-1 expression demonstrated by PE-A histogram of EVs-macrophage populations. (**a**) Two-dimensional plot of TREM-1 PE-H vs violet SSC-H with distribution of EVs spots of size and control allowing the positioning of the region of positivity for TREM-1. (**b**) Histograms show a number of positive events for TREM-1 and PE-A (area).

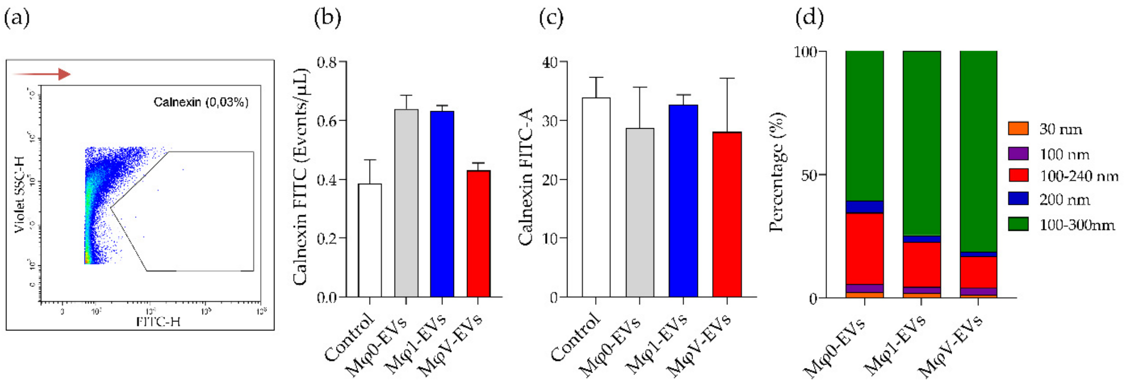

Figure S4. Viral vesicles (MφV-EVs) show a high distribution of nano-sized particles detected by flow cytometry. (**a**) Gating strategy used for detection of calnexin. (**b**,**c**) Calnexin expression. (**d**) Percentage of vesicle size analyzed by the CytoFLEX flow cytometer. Statistical analyzes were performed with a one-way ANOVA test, followed by Tukey's post-test, considering significance at $p < 0.05$.

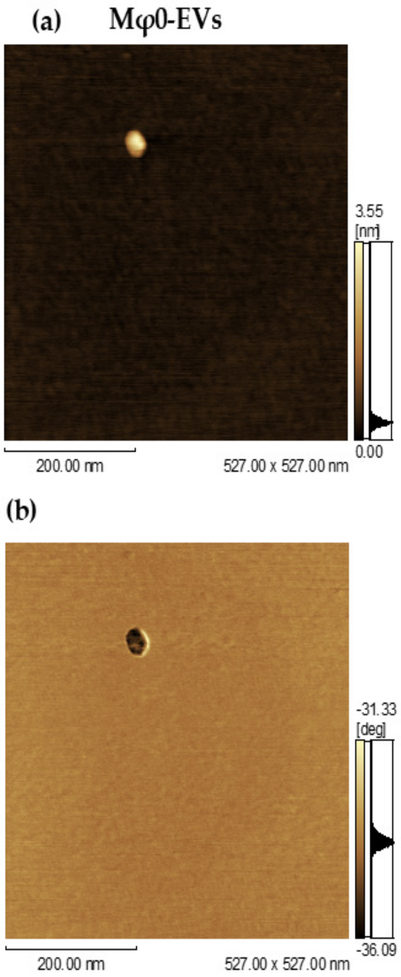

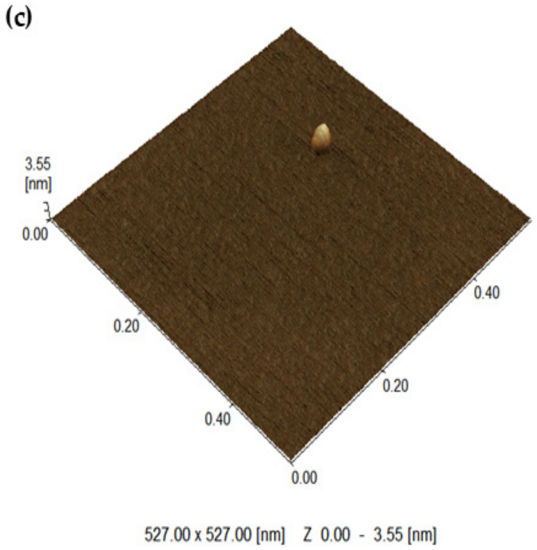

Statistics Value	Maximum Diameter [nm]	Mean Radius [nm]	Ratio Roughness [nm]
Average	57.12±12.25	19.64±4.364	1.336±0.492

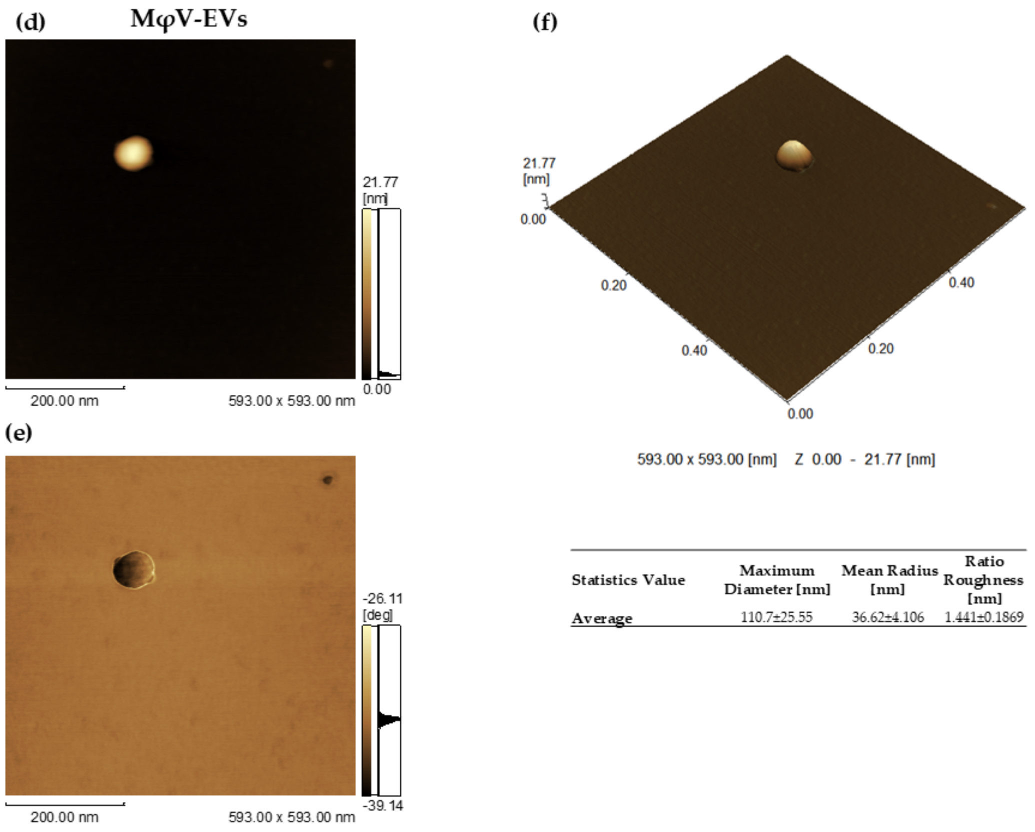

Statistics Value	Maximum Diameter [nm]	Mean Radius [nm]	Ratio Roughness [nm]
Average	110.7±25.55	36.62±4.106	1.441±0.1869

Figure S5. AFM micrographs of Mφ0-EVs and MφV-EVs groups. (**a**,**b**,**d**,**e**) A phase image shows the topology of the vesicles and their protein complexities. (**c**,**f**) The 3D topographic image shows the vesicle topology. The images were obtained using a Shimadzu SPM-9600 scanning probe microscopy (Shimadzu, Japan). For each sample, 10 vesicles were sampled. Abbreviations: AFM, atomic force microscopy.

Table S1. Genes analyzed by qPCR and their respective primers and transcription products for gene expression analysis.

Gene	Forward (5′–3′)	Reverse (5′–3′)
**p16**	GAAGGTCCTCAGACATCCC	AAACTACGAAAGCGGGGTGG
**p53**	CCCTCCTCAGCATCTTATCC	GTACAGTCAGAGCCAACCTCAG
**β-actin**	CCAGCCTTCCTTCCTGGGCAT	AGGGCAATGATCTTGATCTTCATT

Table S2. Raw cycle threshold levels of the panel of miRNAs used in extracellular vesicle profiling.

	Extracellular Vesicles											
**microRNAs**	**Mφ0-EVs**				**Mφ1-EVs**				**MφV-EVs**			
	**1**	**2**	**3**	**Mean**	**1**	**2**	**3**	**Mean**	**1**	**2**	**3**	**Mean**
**miR-21-5p**	27.642	29.227	35.158	30.676	34.838	35.938	-	35.388	26.725	35.774	29.807	30.769
**miR-132**	30.119	28.691	29.923	29.578	31.878	29.345	31.173	30.799	28.314	31.876	29.498	29.896
**miR-155**	31.850	33.399	-	32.625	-	-	34.962	34.962	29.634	36.933	30.264	32.277
**miR-99b**	30.522	28.544	31.197	30.088	36.547	32.748	30.214	33.170	29.237	30.250	30.324	29.937
**Hm/Ms/Rt T1 snRNA**	19.694	21.694	28.549	23.312	30.200	30.155	32.937	21.097	20.573	26.597	21.819	22.966
**RNT43 snoRNA**	29.601	29.778	34.397	31.259	36.732	34.409	-	35.571	29.141	35.152	27.926	30.740
**Negative**	nd	nd	nd	nd	nd	nd	nd	nd	nd	nd	nd	nd
**Geomean**	24.517	24.884	29.844	26.415	33.222	31.425	31.546	32.064	24.525	28.365	25.722	26.204

Table S3. Amount of total protein in Mφ0-EVs and MφV-EVs.

Samples	Protein Amount (μg/μL)
Mφ0-EVs.: lote-1	70.41
Mφ0-EVs.: lote-2	78.96
Mφ0-EVs.: lote-3	101.49
MφV-EVs.: lote-1	47.49
MφV-EVs.: lote-2	75.06
MφV-EVs.: lote-3	81.95
